# Dynamic string‐averaging CQ‐methods for the split feasibility problem with percentage violation constraints arising in radiation therapy treatment planning

**DOI:** 10.1111/itor.12929

**Published:** 2020-12-30

**Authors:** Mark Brooke, Yair Censor, Aviv Gibali

**Affiliations:** ^1^ Department of Oncology University of Oxford Oxford OX3 7DQ UK; ^2^ Department of Mathematics University of Haifa Mt. Carmel Haifa 3498838 Israel; ^3^ Department of Mathematics ORT Braude College Karmiel 2161002 Israel

**Keywords:** string‐averaging, CQ‐algorithm, split feasibility, percentage violation constraints, radiation therapy treatment planning, dose‐volume constraints, common fixed points, cutter operator

## Abstract

We study a feasibility‐seeking problem with percentage violation constraints (PVCs). These are additional constraints that are appended to an existing family of constraints, which single out certain subsets of the existing constraints and declare that up to a specified fraction of the number of constraints in each subset is allowed to be violated by up to a specified percentage of the existing bounds. Our motivation to investigate problems with PVCs comes from the field of radiation therapy treatment planning (RTTP) wherein the fully discretized inverse planning problem is formulated as a split feasibility problem and the PVCs give rise to nonconvex constraints. Following the CQ algorithm of Byrne (2002, *Inverse Problems*, Vol. **18**, pp. 441–53), we develop a string‐averaging CQ‐method that uses only projections onto the individual sets that are half‐spaces represented by linear inequalities. The question of extending our theoretical results to the nonconvex sets case is still open. We describe how our results apply to RTTP and provide a numerical example.

## 1 Introduction

### 1.1 Motivation

In this work, we are motivated by a linear split feasibility problem with percentage violation constraints (PVCs) arising in radiation therapy treatment planning (RTTP). We first provide the background in general terms.

*Inverse RTTP*. This problem, in its fully discretized modeling approach, leads to a linear feasibility problem (LFP). This is a system of linear interval inequalities:

(1) c≤Ax≤b,

wherein the “dose matrix” A is precalculated by techniques called in RTTP “forward calculation” or “forward planning” and the vector x is the unknown vector of “intensities” that, when used in setting up the treatment machine, will realize this specific “treatment plan.” The vectors b and c contain upper and lower bounds on the total dose Ax permitted and required in volume elements (voxels) of sensitive organs/tissues and target areas, respectively, inside the irradiated body. The components of b and c are prescribed by the attending oncologist and given to the treatment planner.

*PVCs*. In general terms, these are additional constraints that are appended to an existing family of constraints. They single out certain subsets of the existing constraints and declare that up to a specified fraction of the number of constraints in each subset is allowed to be violated by up to a specified percentage of the existing bounds. Such PVCs are useful in the inverse problem of RTTP, mentioned above, where they are called “dose‐volume constraints” (DVCs). When the system of linear interval inequalities is inconsistent, that is, there is no solution vector that satisfies all inequalities, the DVCs allow the oncologist and the planner to relax the original constraints in a controlled manner to achieve consistency and find a solution.

*Split feasibility*. PVCs are, by their very nature, integer constraints, which change the feasibility problem to which they are attached from being a continuous feasibility problem into becoming a mixed‐integer feasibility problem. An alternative to the latter is to translate the PVCs into constraints sets that are appended to the original system of linear interval inequalities but are formulated on the vectors Ax, rather than directly on x. This gives rise to a “split feasibility problem,” which is split between two spaces: the space of “intensity vectors” x and the space of “dose vectors” d:=Ax in which A is the operator mapping one space onto the other.

*Nonconvexity*. The constraint sets, which arise from the PVCs, in the space of “dose vectors” are nonconvex sets, but due to their special form enable the calculation of orthogonal projections of points onto them. This opens the door for applying our proposed dynamic string‐averaging (SA) CQ‐method to the RTTP inverse problem with PVCs. Mathematical analysis for the case of nonconvex sets remains an open question. Looking at it from the practical point of view one may consider also alternatives such as reformulating PVCs as $\ell_{1}$‐norm constraints; see, for example, Candès et al. (2008) and Kim et al. (2013).

*Group structure of constraints*. Each row in system (1) represents a constraint on a single voxel. Lumping together constraints of voxels, according to the organ/tissue to which they belong, divides matrix A and the whole system into “groups” of constraints, referred to as “blocks of constraints” in a natural manner. These groups affect the formulation of the split feasibility problem at hand by demanding that the space of intensity vectors x be mapped separately by each group of rows of matrix A into another space of dose vectors d.

### 1.2 Contribution

Motivated by the above, we deal in this paper with the “multiple‐operator split common fixed point problem” (MOSCFPP) defined next.

Problem 1 (**The MOSCFPP**) Let H and K be two real Hilbert spaces, and let r and p be two natural numbers. Let $U_{i}:H\to H$, 1≤i≤p, and $T_{j}:K\to K$,1≤j≤r, be given operators with nonempty fixed point sets $\mathrm{Fix}(U_{i})$ and $\mathrm{Fix}(T_{j})$, respectively. Further, let $A_{j}:H\to K$, for all 1≤j≤r, be given bounded linear operators. In addition, let Φ be another closed and convex subset of H. The MOSCFPP is as follows:

(2) $$\begin{array}{c} \text{Find}\;\text{an}\;x^{*}\in \Phi \;\text{such}\;\text{that}\;x^{*}\in \cap_{i=1}^{p}\mathrm{Fix}\left(U_{i}\right)\;\text{and}, \end{array}$$

(3) $$\begin{array}{cc} \text{for}\;\text{all}\;1 & \leq j\leq r,\;A_{j}x^{*}\in \mathrm{Fix}\left(T_{j}\right). \end{array}$$

This problem formulation unifies several existing “split problem” formulations and, to the best of our knowledge, has not been formulated before. We analyze it and propose a “dynamic SA CQ‐method” to solve it, based on techniques used in some of those earlier formulations. We show in detail how this problem covers and applies to the linear split feasibility problem with DVCs in RTTP. Our convergence results about the dynamic SA CQ‐algorithm presented here rely on convexity assumptions. Therefore, there remains an open question whether our work can be expanded to cover the case of the nonconvex constraints in the space of dose vectors d used in RTTP. Recent work in the field report on strides made in the field of projection methods when the underlying sets are nonconvex; see, for example, Hesse et al. (2014), Bauschke et al. (2014), and Attouch et al. (2013). This encourages us to expand the results presented here in the same way.

### 1.3 Structure of the paper

We begin by briefly reviewing relevant “split problem” formulations that have led to our proposed MOSCFPP and a “dynamic SA CQ‐method” to solve it. Starting from a general formulation of two concurrent inverse problems in different vector spaces connected by a bounded linear operator, we explore the inclusion of multiple convex constraint sets within each vector space. Defining operators that act on each of these sets allows us to formulate equivalent fixed point problems, which naturally leads to our MOSCFPP. We then provide some insight into how one may solve such a problem, using constrained minimization, or successive metric projections as part of a CQ‐type method (Byrne, 2002). These projection methods form the basis of our “dynamic SA CQ‐method,” which is introduced in Section 4. Important mathematical foundations for this method are provided in Section 3, which serve to describe the conditions under which the method converges to a solution in Section 5. Finally, we bring PVCs into our problem formulation (Section 6) and consolidate our work by providing examples of how the MOSCFPP and “dynamic SA CQ‐method” may be applied in RTTP (Section 7). A numerical example is provided on a synthetically created treatment plan, detailed in Section 8.

An important comment must be made here. The introduction of a new mathematical model for an application naturally calls for simulated numerical validation, particularly when a new algorithm is proposed. Here we present a rudimentary numerical example since more complex clinically relevant treatment plans rely heavily on the medical physics context of the RTTP problem. As such, they call for evaluation of the results in the context of the RTTP problem itself and require a dedicated proper background and framework which are outside the scope of this paper. An extensive analysis of the methods presented in this paper, on a number of clinical treatment plans, will be published in an appropriate medical physics journal.

## 2 A brief review of “split problems” formulations and solution methods

The following brief review of “split problems” formulations and solution methods will help put our work in context. The review is nonexhaustive and focuses only on split problems that led to our new formulation that appears in Problem 1. Other split problems such as “the common solution of the split variational inequality problems and fixed point problems” (see, e.g., Lohawech et al., 2018) or “split Nash equilibrium problems for noncooperative strategic games” (see, e.g., Li, 2019) and many others are not included here. The “split inverse problem” (SIP), which was introduced by Censor et al. (2012) (see also Byrne et al., 2012), is formulated as follows.

Problem 2 (**The SIP**) Given are two vector spaces X and Y and a bounded linear operator A:X→Y. In addition, two inverse problems are involved. The first one, denoted by IP${}_{1}$, is formulated in the space X and the second one, denoted by IP${}_{2}$, is formulated in the space Y. The SIP is as follows:

(4) $$\text{Find}\;\text{an}\;x^{*}\in X\;\text{that}\;\text{solves}\;{\text{IP}}_{1}\;\text{such}\;\text{that}\;y^{*}:=Ax^{*}\in Y\;\text{solves}\;{\text{IP}}_{2}.$$

The first published instance of an SIP is the “split convex feasibility problem” (SCFP) of Censor and Elfving (1994), which is formulated as follows.

Problem 3 (**The SCFP**) Let H and K be two real Hilbert spaces. Given are nonempty, closed and convex sets C⊆H and Q⊆K and a bounded linear operator A:H→K. The SCFP is:

(5) $$\text{Find}\;\text{an}\;\;x^{*}\in C\;\text{such}\;\text{that}\;Ax^{*}\in Q.$$

This problem was employed, among others, for solving an inverse problem in intensity‐modulated radiation therapy treatment planning (see Censor et al., 2006; Davidi et al., 2015; Censor et al., 2005). More results regarding the SCFP theory and algorithms, can be found, for example, in Yang (2004); López et al. (2012); Gibali et al. (2018), and the references therein. The SCFP was extended in many directions to Hilbert and Banach spaces formulations. It was extended also to the following “multiple sets split convex feasibility problem” (MSSCFP).

Problem 4 (**The MSSCFP**) Let H and K be two real Hilbert spaces and r and p be two natural numbers. Given are sets $C_{i}$, 1≤i≤p and $Q_{j}$,1≤j≤r, that are closed and convex subsets of H and K, respectively, and a bounded linear operator A:H→K. The MSSCFP is:

(6) $$\text{Find}\;\text{an}\;x^{*}\in \cap_{i=1}^{p}C_{i}\;\text{such}\;\text{that}\;Ax^{*}\in \cap_{j=1}^{r}Q_{j}.$$

Masad and Reich (2007) proposed the “constrained multiple set split convex feasibility problem” (CMSSCFP) which is phrased as follows (see also Latif et al., 2016).

Problem 5 (**The constrained multiple set split convex feasibility problem (CMSSCFP)**) Let H and K be two real Hilbert spaces and r and p be two natural numbers. Given are sets $C_{i}$, 1≤i≤p and $Q_{j}$, 1≤j≤r, which are closed and convex subsets of H and K, respectively, and for 1≤j≤r, given bounded linear operators $A_{j}:H\to K$. In addition let Φ be another closed and convex subset of H. The CMSSCFP is as follows:

(7) $$\text{Find}\;\text{an}\;x^{*}\in \Phi \;\text{such}\;\text{that}\;x^{*}\in \cap_{i=1}^{p}C_{i}\;\text{and}\;A_{j}x^{*}\in Q_{j},\;\text{for}\;1\leq j\leq r.$$

Another extension, due to Censor and Segal (2009), is the following “split common fixed points problem” (SCFPP).

Problem 6 (**The SCFPP**) Let H and K be two real Hilbert spaces and r and p be two natural numbers. Given are operators $U_{i}:H\to H$, 1≤i≤p, and $T_{j}:K\to K$, 1≤j≤r, with nonempty fixed point sets $\mathrm{Fix}(U_{i})$,1≤i≤p and $\mathrm{Fix}(T_{j})$, 1≤j≤r, respectively, and a bounded linear operator A:H→K. The SCFPP is as follows:

(8) $$\text{Find}\;\text{an}\;x^{*}\in \cap_{i=1}^{p}\mathrm{Fix}\left(U_{i}\right)\;\text{such}\;\text{that}\;Ax^{*}\in \cap_{j=1}^{r}\mathrm{Fix}\left(T_{j}\right).$$

Problems 3–6 are SIPs but, more importantly, they are special cases of our MOSCFPP of Problem 1.

Focusing in a telegraphic manner on algorithms for solving some of the above SIPs, we observe that the SCFP of Problem 3 can be reformulated as the constrained minimization problem:

(9) $$\min_{x\in C}\frac{1}{2}{∥P_{Q}\left(Ax\right)-Ax∥}^{2},$$

where $P_{Q}$ is the orthogonal (metric) projection onto Q. Note that the term “orthogonal projection” is commonly used mainly for subspaces while “metric projection” refers to projection onto any kind of sets (see, e.g., Cegielski, 2012, Section 2.2.4). Since the objective function is convex and continuously differentiable with Lipschitz continuous gradients, one can apply the projected gradient method (see, e.g., Goldstein, 1964) and obtain Byrne's well‐known *CQ‐algorithm* (Byrne, 2002). The iterative step of the CQ‐algorithm has the following structure:

(10) $$x^{k+1}=P_{C}\left(x^{k}-\gamma A^{★}\left(Id-P_{Q}\right)Ax^{k}\right),$$

where $A^{★}$ stands for the adjoint ($A^{★}$=$A^{T}$ the transpose, in Euclidean spaces) of A, γ is some positive number, Id is the identity operator, and $P_{C}$ and $P_{Q}$ are the orthogonal projections onto C and Q, respectively. For the MSSCFP of Problem 4, the minimization model considered in Censor et al. (2005), is

(11) $$\min_{x\in R^{M}}\left(\sum_{i=1}^{p}{\text{dist}}^{2}\left(x,C\right)+\sum_{j=1}^{r}{\text{dist}}^{2}\left(Ax,Q\right)\right),$$

leading, for example, to a gradient descent method that has an iterative simultaneous projections nature:

(12) $$x^{k+1}=x^{k}-\gamma \sum_{i=1}^{p}\alpha_{i}\left(Id-P_{C_{i}}\right)x^{k}+\sum_{j=1}^{r}\beta_{j}A^{★}\left(Id-P_{Q_{j}}\right)Ax^{k},$$

where $\gamma \in (0,\frac{2}{L})$ with

(13) $$L:=\sum_{i=1}^{p}\alpha_{i}+\sum_{j=1}^{r}\beta_{j}{∥A∥}_{F}^{2},$$

where ${∥A∥}_{F}^{2}$ is the squared Frobenius norm of A.

Inspired by the above and the work presented in Penfold et al. (2017), we propose in the sequel a “dynamic SA CQ‐method” for solving the MOSCFPP of Problem 1.

## 3 Preliminaries

Through this paper H and K are two real Hilbert spaces and let D⊂H. For every point x∈H, there exists a unique nearest point in D, denoted by $P_{D}\left(x\right)$ such that

(14) $$∥x-P_{D}\left(x\right)∥\leq ∥x-y∥,\;\text{for}\;\text{all}\;y\in D.$$

The operator $P_{D}:H\to H$ is called the *metric projection* onto D.

Definition 1 Let T:H→H be an operator and D⊂H.

(i) The operator T is called Lipschitz continuous on D with constant L>0 if

(15) ∥T(x)−T(y)∥≤L∥x−y∥,forallx,y∈D.

(ii) The operator T is called nonexpansive on D if it is 1‐Lipschitz continuous.

(iii) The fixed point set of T is

(16) Fix(T):={x∈H∣T(x)=x}.

(iv) The operator T is called c‐averaged (c‐av) (Baillon et al., 1978) if there exists a nonexpansive operator N:D→H and a number c∈(0,1) such that

(17) T=(1−c)Id+cN.

In this case we also say that T is c‐av (Byrne, 2004). If two operators $T_{1}$ and $T_{2}$ are $c_{1}$‐av and $c_{2}$‐av, respectively, then their composition $S=T_{1}T_{2}$ is $\left(c_{1}+c_{2}-c_{1}c_{2}\right)$‐av (see Byrne, 2004, Lemma 2.2).

(v) The operator T is called ν ‐inverse strongly monotone (ν‐ism) on D if there exists a number ν>0 such that

(18) $$\left\langle T\left(x\right)-T\left(y\right),x-y\right\rangle \geq {\nu ∥T\left(x\right)-T\left(y\right)∥}^{2},\;\text{for}\;\text{all}\;x,y\in D.$$

(vi) The operator T is called firmly nonexpansive (FNE) on D if

(19) $$\langle T(x)-T(y),x-y\rangle \geq {\left\|T(x)-T(y)\right\|}^{2},\;\text{for}\;\text{all}\;x,y\in D.$$

A useful fact is that T is FNE if and only if its complement Id−T is FNE. Moreover, T is FNE if and only if T is (1/2)‐av (see Goebel and Reich, 1984, Proposition 11.2; Byrne, 2004, Lemma 2.3). In addition, T is averaged if and only if its complement Id−T is ν‐ism for some ν>1/2; (see, e.g., Byrne, 2004, Lemma 2.1).

(vii) The operator T is called quasi‐nonexpansive (QNE)

(20) ∥T(x)−w∥≤∥x−w∥forall(x,w)∈H×Fix(T).

(viii) The operator T is called is called a cutter (also firmly quasi‐ nonexpansive) (T∈T) if Fix(T)≠∅ and

(21) $$\langle T(x)-x,T(x)-w\rangle \leq 0\;\text{for}\;\text{all}\;\left(x,w\right)\in H\times \mathrm{Fix}\left(T\right).$$

(ix) Let λ∈[0,2], the operator $T_{\lambda }:=\left(1-\lambda \right)Id+\lambda T$ is called λ‐relaxation of the operator T. With respect to cutters above it is known that for λ∈[0,1], the λ‐relaxation of a cutter is also a cutter (see, e.g., Cegielski, 2012, Remark 2.1.32).

(x) The operator T is called ρ‐strongly quasi‐nonexpansive (ρ‐SQNE), where ρ≥0, if Fix(T)≠∅ and

(22) ∥T(x)−w∥≤∥x−w∥−ρ∥T(x)−x∥,forall(x,w)∈H×Fix(T).

A useful fact is that a family of SQNE operators with non‐empty intersection of fixed point sets is closed under composition and convex combination (see, e.g., Cegielski, 2012, Corollary 2.1.47).

(xi) The operator T is called is called demi‐closed at y∈H if for any sequence ${\left\{x^{k}\right\}}_{k=0}^{\infty }$ in D such that $x^{k}\to \bar{x}\in D$ and $T(x^{k})\to y$, we have $T(\bar{x})=y$.

Next we recall the well‐known *demi‐closedness principle* (Browder, 1965).

Lemma 1 Let H be a Hilbert space, D a closed and convex subset of H, and N:D→H a nonexpansive operator. Then Id−N (Id is the identity operator on H) is demi‐closed at y∈H.

Let A:H→K be a bounded linear operator with ∥A∥>0, and C⊆H and Q⊆K be nonempty, closed, and convex sets. The operator V:H→H which is defined by

(23) $$V:=Id-\frac{1}{{∥A∥}^{2}}A^{★}\left(Id-P_{Q}\right)A$$

is called a *Landweber operator* and U:H→H defined by

(24) $$U:=P_{C}V$$

is called a *projected Landweber operator* with V as in (23); see, for example, Cegielski (2012, 2015, 2016).

In the general case where T:H→H is quasi‐nonexpansive and A:H→K is a bounded and linear operator with ∥A∥>0, a so‐called *Landweber‐type operator* (see, e.g., Cegielski, 2016) is defined by

(25) $$V:=Id-\frac{1}{{∥A∥}^{2}}A^{★}\left(Id-T\right)A.$$

Note that (23) is a special case of (25), since $P_{Q}$ is FNE, thus, quasi‐nonexpansive.

## 4 The dynamic string‐averaging CQ‐method

In this section we present our “dynamic SA CQ‐method” for solving the MOSCFPP of Problem 1. It is actually an algorithmic scheme which encompasses many specific algorithms that are obtained from it by different choices of strings and weights. First, for all j=1,2,…,r, construct from the given data of Problem 1, the operators $V_{j}:H\to H$ defined by

(26) $$V_{j}:=Id-\gamma_{j}A_{j}^{★}\left(Id-T_{j}\right)A_{j},$$

where $\gamma_{j}\in \left(0,\frac{1}{L_{j}}\right)$, $L_{j}={∥A_{j}∥}^{2}$. For quasi‐nonexpansive $T_{j}$ this definition coincides with that of “Landweber‐type operators related to $T_{j}$” of Cegielski (2016, Definition 2) with a relaxation of $\gamma_{j}$.

For simplicity, and without loss of generality, we assume that r=p in Problem 1. This is not restrictive since if r<p we will define $T_{j}:=Id$ for r+1≤j≤p, and if p<r we will define $U_{i}:=Id$ for p+1≤i≤r, which, in both cases, will not make any difference to the formulation of Problem 1.

Define Γ:={1,2,⋯,p} and for each i∈Γ define the operator $R_{i}:H\to H$ by$R_{i}:=U_{i}V_{i}$. An *index vector* is a vector $t=(t_{1},t_{2},\cdots ,t_{q})$ such that $t_{i}\in \Gamma$ for all i=1,2,⋯,q. For a given index vector $t=(t_{1},t_{2},\cdots ,t_{q})$ we denote its *length* by ℓ(t):=q, and define the operator Z[t] as the product of the individual operators $R_{i}$ whose indices appear in the index vector t, namely,

(27) $$Z\left[t\right]:=R_{t_{\ell (t)}}R_{t_{\ell (t)-1}}\cdots R_{t_{1}},$$

and call it a *string operator*. A finite set Θ of index vectors is called *fit* if for each i∈Γ, there exists a vector $t=(t_{1},t_{2},\cdots ,t_{q})\in \Theta$ such that $t_{s}=i$ for some s∈Γ.

Denote by M the collection of all pairs (Θ,w), where Θ is a fit finite set of index vectors and

(28) $$w:\Theta \to \left(0,\infty \right)\;\text{is}\;\text{such}\;\text{that}\;\sum_{t\in \Theta }w\left(t\right)=1.$$

For any (Θ,w)∈M define the convex combination of the end points of all strings defined by members of Θ by

(29) $$\Psi_{\Theta ,w}\left(x\right):=\sum_{t\in \Theta }w\left(t\right)Z\left[t\right]\left(x\right),\;x\in H.$$

We fix a number Δ∈(0,1/p) and an integer $\bar{q}\geq p$ and denote by $M_{*}\equiv M_{*}\left(\Delta ,\bar{q}\right)$ the set of all (Θ,w)∈M such that the lengths of the strings are bounded and the weights are all bounded away from zero, namely,

(30) $$M_{*}:=\left\{\left(\Theta ,w\right)\in M|\;\ell \left(t\right)\leq \bar{q}\;\text{and}\;w\left(t\right)\geq \Delta \;\text{for}\;\text{all}\;t\in \Theta \right\}.$$

The dynamic SA CQ‐method with variable strings and variable weights is described by the following iterative process.

Algorithm 1 **The dynamic SA CQ‐method with variable strings and variable weights**

**Table:** 

**Initialization**: Select an arbitrary $x^{0}\in H$,
**Iterative step**: Given a current iteration vector $x^{k}$ pick a pair $\left(\Theta_{k},w_{k}\right)\in M_{*}$ and calculate the next iteration vector by $x^{k+1}=\Psi_{\Theta_{k},w_{k}}\left(x^{k}\right).\;\;\;\;\;\;\;\;\;\;\;\;\;\;\;\;\;\;\;\;\left(31\right)$

The iterative step of (31) amounts to calculating, for all $t\in \Theta_{k}$, the strings' end points

(32) $$Z\left[t\right]\left(x^{k}\right)=R_{i_{\ell (t)}^{t}}\cdots R_{i_{2}^{t}}R_{i_{1}^{t}}\left(x^{k}\right),$$

and then calculating

(33) $$x^{k+1}=\sum_{t\in \Theta_{k}}w_{k}\left(t\right)Z\left[t\right]\left(x^{k}\right).$$

This algorithmic scheme applies to $x^{k}$ successively the operators $R_{i}:=U_{i}V_{i}$ whose indices belong to the string t. This can be done in parallel for all strings and then the end points of all strings are convexly combined, with weights that may vary from iteration to iteration, to form the next iterate $x^{k+1}$. This is indeed an algorithm provided that the operators ${\left\{R_{i}\right\}}_{i=1}^{p}$ all have algorithmic implementations. In this framework, we get a *sequential algorithm* by allowing a single string created by the index vector t=Γ and a *simultaneous algorithm* by the choice of p different strings of length one each containing one element of Γ. Intermediate structures are possible by judicious choices of strings and weights.

## 5 Convergence

Next we prove the equivalence between Problem 1 and a common fixed point problem which is not split, give a description of Fix$\left(V_{j}\right)$, and state a property of $V_{j}$.

Lemma 2 Denote the solution set of Problem 1 by Ω and assume that it is nonempty. Then, for $V_{j}$ as in (26),

(i) $x^{*}\in \Omega$ if and only if $x^{*}$ solves the common fixed point problem:

(34) $$\text{Find}\;x^{*}\in \left(\cap_{i=1}^{p}\text{Fix}\left(U_{i}\right)\right)\cap \left(\cap_{j=1}^{r}\text{Fix}\left(V_{j}\right)\right);$$

(ii) for all j=1,2,…,r:

(35) $$\text{Fix}\;\left(V_{j}\right)=\left\{x\in H|A_{j}x\in \text{Fix}\;\left(T_{j}\right)\right\}=A_{j}^{-1}\left(\text{Fix}\;\left(T_{j}\right)\right),$$

where $A_{j}^{-1}$ denotes here the inverse image (preimage) of $A_{j}$, that is, $A_{j}^{-1}:K\to H$ and for any y∈K, $A_{j}^{-1}\left(y\right):=\left\{x\in H|A_{j}x=y\right\}$;

(iii) if, in addition, all operators $T_{j}$ are cutters then all $V_{j}$ are cutters (i.e., are 1‐SQNE);

(iv) if $T_{j}$ is ρ‐SQNE, $A_{j}\cap \text{Fix}T_{j}\neq \emptyset$ (here we refer to $A_{j}$ as the image set of $A_{j}$) and satisfies the demi‐closedness principle then $V_{j}$ also satisfies the demi‐closedness principle.

(i) We need to show only that

(36) $$x^{*}\in \cap_{j=1}^{r}\text{Fix}\left(V_{j}\right)\Leftrightarrow A_{j}x^{*}\in \text{Fix}\;\left(T_{j}\right)\;\text{for}\;\text{all}\;j=1,2,\text{…},r.$$

Indeed, for any j=1,2,…,r,

(37) $$\begin{array}{c} A_{j}x^{*}\in \text{Fix}\;\left(T_{j}\right)\Leftrightarrow A_{j}x^{*}-T_{j}A_{j}x^{*}=0\\ \;\Leftrightarrow A_{j}^{★}\left(Id-T_{j}\right)A_{j}x^{*}=A_{j}^{★}0\;\Leftrightarrow -\gamma_{j}A_{j}^{★}\left(Id-T_{j}\right)A_{j}x^{*}=0\\ \;\Leftrightarrow x^{*}-\gamma_{j}A_{j}^{★}\left(Id-T_{j}\right)A_{j}x^{*}=x^{*}\Leftrightarrow x^{*}\in \text{Fix}\left(V_{j}\right). \end{array}$$

(ii) Follows from (37).

(iii) To show that $V_{j}$ is a cutter take $w\in \text{Fix}(V_{j})$, $\gamma_{j}\in \left(0,\frac{1}{L_{j}}\right)$ and ξ∈H.

(38) $$\begin{array}{ccc} \frac{1}{\gamma_{j}}\langle w-V_{j}\left(\xi \right),\xi -V_{j}\left(\xi \right)\rangle & = & \langle w-\xi -\gamma_{j}A_{j}^{★}\left(T_{j}-Id\right)A_{j}\xi ,A_{j}^{★}\left(Id-T_{j}\right)A_{j}\xi \rangle \\ & = & \langle w-\xi ,A_{j}^{★}\left(Id-T_{j}\right)A_{j}\xi \rangle +\gamma_{j}{∥A_{j}^{★}\left(Id-T_{j}\right)A_{j}\xi ∥}^{2}\\ & = & \langle A_{j}w-A_{j}\xi ,\left(Id-T_{j}\right)A_{j}\xi \rangle +\gamma_{j}{∥A_{j}^{★}\left(Id-T_{j}\right)A_{j}\xi ∥}^{2}\\ & = & \langle A_{j}w-T_{j}\left(A_{j}\xi \right),\left(Id-T_{j}\right)A_{j}\xi \rangle +\gamma_{j}{∥A_{j}^{★}\left(Id-T_{j}\right)A_{j}\xi ∥}^{2}\\ & & -\;∥\left(Id-T_{j}\right)A_{j}{\xi ∥}^{2}. \end{array}$$

Since $T_{j}$ is a cutter and $A_{j}w\in \text{Fix}\;\left(T_{j}\right)$, we have

(39) $$\langle A_{j}w-T_{j}\left(A_{j}\xi \right),\left(Id-T_{j}\right)A_{j}\xi \rangle \leq 0.$$

Also,

(40) $$\gamma_{j}∥A_{j}^{★}\left(Id-T_{j}\right)A_{j}{\xi ∥}^{2}\leq \gamma_{j}∥A_{j}{∥}^{2}∥\left(Id-T_{j}\right)A_{j}{\xi ∥}^{2}\leq {∥\left(Id-T_{j}\right)A_{j}\xi ∥}^{2},$$

for all $\gamma_{j}\in \left(0,1/L_{j}\right)$. Using the above we get that

(41) $$\langle w-V_{j}\left(\xi \right),\xi -V_{j}\left(\xi \right)\rangle \leq 0,$$

which proves that $V_{j}$ is a cutter.

(iv) Proved in Cegielski (2016, Theorem 8(iv)).□

The special case where in Problem 1 there is only one operator A:H→K and (3) is replaced by

(42) $$\text{for}\;\text{all}\;1\leq j\leq r,\;Ax^{*}\in \text{Fix}\;\left(T_{j}\right),$$

which amounts to $Ax^{*}\in \cap_{j=1}^{r}$Fix $\left(T_{j}\right)$ was treated in the literature (see, e.g., Wang and Xu, 2011; Cegielski, 2015, 2016). The extensions to our more general case, necessitated by the application to RTTP at hand, follow the patterns in those earlier papers. In our convergence analysis, we rely on the convergence result of Reich and Zalas (2016, Theorem 4.1) who, motivated by Censor and Tom (2003, Algorithm 3.3), invented and investigated the “modular SA (MSA) method” (Reich and Zalas, 2016, Procedure 1.1).

For the convenience of the readers we quote next in full details Procedure 1.1 and Theorem 4.1 of Reich and Zalas (2016). We adhere to the original notations of Reich and Zalas and later identify them with the notations of our work. Let $U_{i}:H\to H$ be a finite family of quasi‐nonexpansive mappings where i∈I:={1,2,⋯,M} and define $U_{0}:=Id$. The problem under investigation is the common fixed point problem of finding an $x\in C:=\cap_{i\in I}$Fix$\left(U_{i}\right)$. The algorithmic scheme is

(43) $$x^{0}\in H,\;\;x^{k+1}=T_{k}x^{k},$$

where the operator $T_{k}$ depends on a chosen subset of the input operators $U_{i}$.

Reich and Zalas proposed Procedure 1.1 for constructing operators $T_{k}$ (called “modules”) is as follows. Fix N∈N, for n=1,2,…,N; let ε∈(0,1) be a fixed parameter; define modules $V_{n}:=U_{-n}$ for all n=−M,…,0. For n=1,2,…,N define modules $V_{n}$ by choosing one of the following cases:

(a) Relaxation: Fix a singleton $J_{n}=\left\{j_{n}\right\}\subseteq \left\{-M,\text{…},0\right\}$ and a relaxation $\alpha_{n}\in \left[\varepsilon ,2-\varepsilon \right]$, and set

(44) $$V_{n}:=Id+\alpha_{n}\left(V_{j_{n}}-Id\right).$$

(b) Convex combination: Fix a nonempty subset $J_{n}\subseteq \left\{-M,\text{…},n-1\right\}$ and weights $\omega_{j,n}\in \left[\varepsilon ,1-\varepsilon \right]$ satisfying $\sum_{j\in J_{n}}\omega_{j,n}=1$, and set

(45) $$V_{n}:=\sum_{j\in J_{n}}\omega_{j,n}V_{j}.$$

(c) Composition: Fix a “string” $J_{n}\subseteq \left\{-M,\text{…},n-1\right\}$ with length less than M+n and set

(46) $$V_{n}:=\Pi_{j\in J_{n}}V_{j}.$$

Using the above MSA procedure of Reich and Zalas, by preforming $N_{k}$ steps with parameter $\varepsilon_{k}>0$, $T_{k}$ is defined as the last module from the pool, that is, $T_{k}:=V_{N_{k}}^{k}$. Such constructions of the operators $T_{k}$ lead to various combination schemes such as sequential, convex combination, and composition. An SA scheme that is relevant to our method here is obtained by taking a convex combination of multiple compositions, as in Reich and Zalas (2016, Equation (1.12)).

Reich and Zalas Theorem 4.1 is quoted next.

Theorem 2 Let ${\left\{x^{k}\right\}}_{k=0}^{\infty }$ be a sequence generated by the iterative method

(47) $$x^{0}\in H,\;x^{k+1}=T_{k}\left(x^{k}\right)$$

and assume that

(i) each operator $U_{i}$, i∈I is a cutter;

(ii) $I\subseteq I_{k}\cup I_{k+1}\cup \cdots \cup I_{k+s-1}$, for each k=0,1,2,…, and some s≥M−1;

(iii) the sequence ${\left\{N_{k}\right\}}_{k=0}^{\infty }$ is bounded.

If, for each i∈I, the operator $U_{i}$ satisfies Opial's demi‐closedness principle then the sequence ${\left\{x^{k}\right\}}_{k=0}^{\infty }$ converges weakly to some point in C.

If, for each i∈I, the operator $U_{i}$ is approximately shrinking and the family $C:=\{\mathrm{Fix}\;U_{i}|i\in I\}$ is boundedly regular then the sequence ${\left\{x^{k}\right\}}_{k=0}^{\infty }$ converges strongly to some point in C.

Our convergence theorem for the dynamic SA CQ‐method now follows.

Theorem 3 Let p≥1 be an integer and suppose that Problem 1 with r=p has a nonempty solution set Ω. Let ${\left\{U_{i}\right\}}_{i=1}^{p}$ and ${\left\{T_{i}\right\}}_{i=1}^{p}$ be cutters on Hilbert spaces H and K, respectively. Further assume that $U_{i}-Id$ and $T_{i}-Id$ are demi‐closed at zero for all i. Then any sequence ${\left\{x^{k}\right\}}_{k=0}^{\infty }$, generated by Algorithm 1 with $R_{i}:=U_{i}V_{i}$ for all i, where $V_{i}$ are defined as in (26), converges weakly to a point $x^{*}\in \Omega$.

First, we identify the notations in our work with those in Reich and Zalas (2016).

(i) The operators ${\left\{U_{i}\right\}}_{i=1}^{M}$ of Reich and Zalas (2016, Theorem 4.1) are our ${\left\{R_{i}\right\}}_{i=1}^{p}$ where $R_{i}:=U_{i}V_{i}$ as described in the beginning of Section 4.

(ii) Our operators $\Psi_{\Theta_{k},w_{k}}$ (31) are identified with the algorithmic operators $T_{k}$ of Equation (1.12) in Reich and Zalas (2016).

(iii) Our operators ${\left\{U_{i}\right\}}_{i=1}^{p}$ and ${\left\{T_{i}\right\}}_{i=1}^{p}$ are assumed to be cutters, then so are also ${\left\{V_{i}\right\}}_{i=1}^{p}$, by Lemma 2(iii). Hence, the composition operators $R_{i}:=U_{i}V_{i}$ are ρ‐SQNE for all i and, therefore, also our $\Psi_{\Theta_{k},w_{k}}$ are ρ‐SQNE for all k.

(iv) We assume that our $U_{i}-Id$ and $T_{i}-Id$ are demi‐closed at zero for all i, therefore, by Lemma 2(iv), $V_{i}-Id$ are also demi‐closed at zero. So, our operators $R_{i}=U_{i}V_{i}$, as composition of demi‐closed operators, are demi‐closed, see for example (Cegielski, 2015, Theorem 4.2). Our operators $R_{i}=U_{i}V_{i}$ are identified with ${\left\{U_{i}\right\}}_{i=1}^{M}$ of Reich and Zalas (2016).

Next, we show that our dynamic SA CQ‐method fits into the MSA (Reich and Zalas, 2016, Procedure 1.1) and that the assumptions of Reich and Zalas (2016, Theorem 4.1) hold.

Since we identify our $\Psi_{\Theta_{k},w_{k}}$ from (31) with the right‐hand side of Equation (1.12) of Reich and Zalas (2016) (being careful with regard to the duplicity of symbols that represent different things in that work and here), Algorithm 1 can be represented by the iterative process of Equation (1.2), or Equation (4.2), of Reich and Zalas (2016).

Next we show the validity of the assumptions needed by Reich and Zalas (2016, Theorem 4.1).

Assumption (i) of Reich and Zalas (2016, Theorem 4.1): The operators ${\left\{U_{i}\right\}}_{i=1}^{M}$ of Reich and Zalas (2016, Theorem 4.1) are our $R_{i}:=U_{i}V_{i}$. Although our $R_{i}$ are not necessarily cutters, the arguments in the proof of Reich and Zalas (2016, Theorem 4.1) are based on the strongly quasi‐nonexpansiveness of the operators $T_{k}$ there (our $\Psi_{\Theta_{k},w_{k}}$) and by Lemma 2(iii), our operators ${\left\{V_{i}\right\}}_{i=1}^{p}$ (defined in (26)) are cutters and this together with the assumption on our ${\left\{U_{i}\right\}}_{i=1}^{p}$ and ${\left\{T_{i}\right\}}_{i=1}^{p}$, yields that the composition operators $R_{i}:=U_{i}V_{i}$ are ρ‐SQNE for all i and, thus, so are also our $\Psi_{\Theta_{k},w_{k}}$.

Assumptions (ii) + (iii) of Reich and Zalas (2016, Theorem 4.1): Since the construction of the operators $\Psi_{\Theta_{k},w_{k}}$ is based on $M_{*}$ (30) which mandates a fit Θ, it guarantees that every index i∈Γ appears in the construction of $\Psi_{\Theta_{k},w_{k}}$ for all k>0, thus, Assumption (ii) in Reich and Zalas (2016, Theorem 4.1) holds. Following the same reasoning, it is clear that the number of steps $N_{k}$, defined in the MSA (Reich and Zalas, 2016, Procedure 1.1), is bounded.

The weak convergence part of the proof of Reich and Zalas (2016, Theorem 4.1) requires that all (their) ${\left\{U_{i}\right\}}_{i=1}^{M}$ satisfy Opial's demi‐closedness principle (i.e., that $U_{i}-Id$ are demi‐closed at zero). In our case, we assume that $U_{i}-Id$ and $T_{i}-Id$ are demi‐closed at zero for all i. By Lemma 2(iv) above $V_{i}-Id$ are also demi‐closed at zero. So, we identify ${\left\{U_{i}\right\}}_{i=1}^{M}$ of Reich and Zalas (2016) with our $U_{i}$s and $V_{i}$s and construct first the operators $R_{i}=U_{i}V_{i}$, and then use them as the building bricks of the algorithmic operators $\Psi_{\Theta_{k},w_{k}}$.

Observe that in our proposed dynamic SA scheme the weights are chosen, in every iteration k, so that $\left(\Theta_{k},w_{k}\right)\in M_{*}$ (see the iterative step of Algorithm 1). This requires, according to (30), that w(t)≥Δforallt∈Θ, where Δ∈(0,1/p) is a fixed positive number. Therefore, for any t it must hold that $\sum_{k=0}^{\infty }w_{k}\left(t\right)=\infty$, meaning that we “visit” every operator infinitely many times. This fully coincides with the assumption in (Reich and Zalas, 2016) that $w_{k}\left(i\right)\in \left[\varepsilon ,1-\varepsilon \right]$ for some ε>0 which implies that $\sum_{k=0}^{\infty }w_{k}\left(i\right)=\infty$ for all i, in their notation.

Thus, the desired result is obtained.□

Remark 1 (i) If one assumes that the $T_{j}$ operators are FNE, then similar arguments as in the proof of Moudafi (2011, Theorem 3.1) show that the $V_{j}$ operators are also averaged and then Reich and Zalas (2016, Theorem 4.1) can be adjusted to hold for averaged operators.

(ii) It is possible to propose inexact versions of Algorithm 1 following Reich and Zalas (2016, Theorem 4.5) and Combettes' “almost cyclic sequential algorithm” (Combettes, 2001, Algorithm 6.1).

(iii) Our work can be extended to cover also underrelaxed operators, that is, by defining $R_{i}:={\left(U_{i}\right)}_{\lambda }{\left(V_{i}\right)}_{\delta }$ for λ,δ∈[0,1]. This is allowed due the fact that if an operator is firmly quasi‐nonexpansive, then so is its relaxation.

(iv) Reich and Zalas (2016, Theorem 4.1) also includes a strong convergence part under some additional assumptions on their operators ${\left\{U_{i}\right\}}_{i=1}^{M}$. It is possible to adjust this theorem for our case as well.

(v) We proposed here a general scheme that allows dynamic SA; the closest CQ variant appears in the work of Wang and Xu (2011, Theorem 3.1) where only sequential, cyclically controlled, iterations are allowed.

(vi) For the case of a two‐set nonconvex feasibility problem (non‐CFP), Attouch et al. (2013, Theorem 5.3) propose a CQ variant but without a relaxation and if more than two nonconvex sets are allowed, then a fully simultaneous method is obtained.

## 6 Percentage violation constraints arising in radiation therapy treatment planning

### 6.1 Transforming problems with a PVC

Given p closed convex subsets $\Omega_{1},\Omega_{2},\cdots ,\Omega_{p}\subseteq R^{n}$ of the n‐dimensional Euclidean space $R^{n}$, expressed as level sets:

(48) $$\Omega_{j}=\left\{x\in R^{n}|f_{j}\left(x\right)\leq v_{j}\right\},\;\text{for}\;\text{all}\;j\in J:=\left\{1,2,\text{…},p\right\},$$

where $f_{j}:R^{n}\to R$ are convex functions and $v_{j}$ are some given real numbers, the CFP is to find a point $x^{*}\in \Omega :=\cap_{j\in J}\Omega_{j}$. If Ω=∅ then the CFP is said to be inconsistent.

Problem 7 (**CFP with a PVC (CFP + PVC)**) Consider p closed convex subsets $\Omega_{1},\Omega_{2},\cdots ,\Omega_{p}\subseteq R^{n}$ of the n‐dimensional Euclidean space $R^{n}$, expressed as level sets according to (48). Let 0≤α≤1 and 0<β<1 be two given real numbers. The CFP+PVC is as follows:

Find an $x^{*}\in R^{n}$ such that $x^{*}\in \cap_{j=1}^{p}\Omega_{j}$ and in up to a fraction α (i.e., 100α%) of the total number of inequalities in (48) the bounds $v_{j}$ may be potentially violated by up to a fraction β (i.e., 100β%) of their values.

A PVC is an integer constraint by its nature. It changes the CFP (48) to which it is attached from being a continuous feasibility problem into becoming a mixed‐integer feasibility problem. Denoting the inner product of two vectors in $R^{n}$ by $\left\langle a,b\right\rangle :=\sum_{i=1}^{n}a_{i}b_{i}$, the LFP with PVC (LFP + PVC) is the following special case of Problem 7.

Problem 8 (**LFP with a PVC (LFP + PVC)**) This is similar to Problem 7 with $f_{j}$, for j=1,2,…,p, in (48) being linear functions, meaning that the sets $\Omega_{j}$ are half‐spaces:

(49) $$\Omega_{j}=\left\{x\in R^{n}|\langle a^{j},x\rangle \leq b_{j}\right\},\;\text{for}\;\text{all}\;j\in J,$$

for a set of given vectors $a^{j}\in R^{n}$ and $b_{j}$ some given real numbers.

Our tool to “translate” the mixed‐integer LFP + PVC into a “continuous” one is the notion of sparsity norm, called elsewhere the zero‐norm, of a vector $x\in R^{n}$ which counts the number of nonzero entries of x, that is,

(50) $${∥x∥}_{0}:=\left|\{x_{i}|x_{i}\neq 0\}\right|,$$

where ∣·∣ denotes the cardinality, that is, the number of elements, of a set. This notion has been recently used for various purposes in compressed sensing, machine learning and more. The rectifier (or “positive ramp operation”) on a vector $x\in R^{n}$ means that, for all i=1,2,…,n:

(51) $${\left(x_{+}\right)}_{i}:=\max\left(0,x_{i}\right)=\left\{\begin{array}{c} x_{i},\;\text{if}\;x_{i}>0,\\ 0,\;\text{if}\;x_{i}\leq 0. \end{array}\right.$$

Obviously, $x_{+}$ is always a component‐wise nonnegative vector. Hence, $∥x_{+}{∥}_{0}$ counts the number of positive entries of x and is defined by

(52) $$∥x_{+}{∥}_{0}:=\left|\left\{x_{i}|x_{i}>0\right\}\right|.$$

We translate the LFP + PVC to the following.

Problem 9 (**Translated problem of LFP + PVC (for LFP with upper bounds)**) For the data of Problem 8, let $A\in R^{p\times n}$ be the matrix whose columns are formed by the vectors $a^{j}$ and let $b\in R^{p}$ be the column vector consisting of the values $b_{j}$, for all j∈J. The translated problem of LFP+PVC (for LFP with upper bounds) is as follows:

(53) $$\begin{array}{c} \text{Find}\;\text{an}\;x^{*}\in R^{n}\;\text{such}\;\text{that}\;\langle a^{j},x^{*}\rangle \leq \left(1+\beta \right)b_{j}, \end{array}$$

(54) $$\begin{array}{c} \text{for}\;\text{all}\;j\in J,\;\text{and}\;∥{\left(Ax^{*}-b\right)}_{+}{∥}_{0}\leq \alpha p. \end{array}$$

The number of the violations in (53) is $∥{\left(Ax^{*}-b\right)}_{+}{∥}_{0}$ and $∥{\left(Ax^{*}-b\right)}_{+}{∥}_{0}\leq \alpha p$ guarantees that the number of violations of up to β in the original row inequalities remains at bay as demanded. This is a split feasibility problem between the space $R^{n}$ and the space $R^{p}$ with the matrix A mapping the first to the latter. The constraints in $R^{n}$ are linear (thus convex) but the constraint

(55) $$x^{*}\in S:=\{y\in R^{p}{|∥\left(y-b\right)}_{+}{∥}_{0}\leq \alpha p\}$$

is not convex. This makes Problem 9 similar in structure to, but not identical with, Problem 3.

Similarly, if the linear inequalities in Problem 9 are in an opposite direction, i.e., of the form $c_{j}\leq \left\langle a^{j},x\right\rangle$, for all j∈J, then the translated problem of LFP+PVC will be as follows.

Problem 10 (**Translated problem of LFP + PVC (for LFP with lower bounds)**) For the data of Problem 8, let $A\in R^{p\times n}$ be the matrix whose columns are formed by the vectors $a^{j}$ and let $c\in R^{p}$ be the column vector consisting of the values $c_{j}$, for all j∈J. The translated problem of LFP+PVC (for LFP with lower bounds) is as follows:

(56) $$\begin{array}{c} \text{Find}\;\text{an}\;x^{*}\in R^{n}\;\text{such}\;\text{that}\;\left(1-\beta \right)c_{j}\leq \langle a^{j},x^{*}\rangle , \end{array}$$

(57) $$\begin{array}{c} \text{for}\;\text{all}\;j\in J,\;\text{and}\;∥{\left(c-Ax^{*}\right)}_{+}{∥}_{0}\leq \alpha p. \end{array}$$

This is also a split feasibility problem between space $R^{n}$ and space $R^{p}$ with matrix A mapping the first to the latter. The constraints in $R^{n}$ are linear (thus convex) but the constraint

(58) $$x^{*}\in T:=\{y\in R^{p}{|∥\left(c-y\right)}_{+}{∥}_{0}\leq \alpha p\}$$

is again not convex.

### 6.2 Translated block LFP + PVC

Consider an m×n matrix A divided into blocks $A_{\ell }$, for ℓ=1,2,…,Γ, with each block forming an $m_{\ell }\times n$ matrix and $\sum_{{}_{\ell =1}}^{\Gamma }m_{\ell }=m$. Further, the blocks are assumed to give rise to block‐wise LFPs of the two kinds; those with upper bounds, say for ℓ=1,2,…,p, and those with lower bounds, say for ℓ=p+1,p+2,…,p+r. PVCs are imposed on each block separately with parameters $\alpha_{\ell }$ and $\beta_{\ell }$, respectively, for all ℓ=1,2,…,Γ. The original block‐LFP prior to imposing the PVCs is

(59) $$\begin{array}{cc} A_{\ell }x\leq b^{\ell }, & \text{for}\;\text{all}\;\ell =1,2,\text{…},p,\\ c^{\ell }\leq A_{\ell }x, & \text{for}\;\text{all}\;\ell =p+1,p+2,\text{…},p+r. \end{array}$$

Such constraints will be termed “hard dose constraints” (HDCs). After imposing the PVCs and translating the systems according to the principles of Problems 9 and 10 we obtain the translated problem of LFP + PVC for blocks.

Problem 11 (**Translated problem of LFP + PVC for blocks**) Find an $x^{*}\in R^{n}$ such that

(60) $$\begin{array}{cc} A_{\ell }x^{*}\leq \left(1+\beta_{\ell }\right)b^{\ell }, & \text{for}\;\text{all}\;\ell =1,2,\text{…},p,\\ \left(1-\beta_{\ell }\right)c^{\ell }\leq A_{\ell }x^{*}, & \text{for}\;\text{all}\;\ell =p+1,p+2,\text{…},p+r,\\ ∥{\left(A_{\ell }x^{*}-b^{\ell }\right)}_{+}{∥}_{0}\leq \alpha_{\ell }m_{\ell }, & \text{for}\;\text{all}\;\ell =1,2,\text{…},p,\\ ∥{\left(c^{\ell }-A_{\ell }x^{*}\right)}_{+}{∥}_{0}\leq \alpha_{\ell }m_{\ell }, & \text{for}\;\text{all}\;\ell =p+1,p+2,\text{…},p+r. \end{array}$$

This is a split feasibility problem between the space $R^{n}$ and the space $R^{m}$ but with a structure similar to Problem 5 where, for ℓ=1,2,…,Γ, each $A_{\ell }$ maps $R^{n}$ to $R^{m_{\ell }}$. Again, it is not identical with Problem 5 because here the constraints in $R^{m_{\ell }}$, for ℓ=1,2,…,Γ, are not convex. . Although Problem 11 defines an upper PVC on exactly p blocks and a lower PVC on exactly r blocks, we can, without loss of generality, choose to define PVCs only on a subset of these blocks. For blocks without a PVC, the problem reverts to a standard LFP.

## 7 Application to radiation therapy treatment planning

The process of planning a radiotherapy treatment plan involves a physician providing dose prescriptions that geometrically constrain the distribution of dose deposited in the patient. Choosing the appropriate nonnegative weights of many individual beamlet dose kernels to achieve these prescriptions as best as possible is posed as a SIP. We focus, for our purposes, on constraining the problem with upper and lower dose bounds, and DVCs, which we more generally refer to as PVCs in this work. DVCs allow dose levels in a specified proportion of a structure to fall short of, or exceed, their prescriptions by a specified amount. They largely serve to allow more flexibility in the solution space.

Problem 11 describes the split feasibility problem as it applies in the context of RTTP. Each block represents a defined geometrical structure in the patient, which is classified either as an *avoidance structure* or a *target volume*. An example of an avoidance structure is an organ at risk, in which one wishes to deposit minimal dose. An example of a target structure is the planning target volume, to which a sufficient dose is prescribed to destroy the tumoral tissue. If there are p avoidance structures, any number of blocks in {1,2,…,p} can have lower PVCs applied. Similarly, if there are r target volumes then any number of blocks in {p+1,p+2,…,p+r} can have an upper PVC applied.

This problem can be formulated as the MOSCFPP described in Problem 1 as follows. For the data of Problem 11, define $\bar{\Gamma }\subseteq \left\{1,2,\text{…},p+r\right\}$ and for all $i=1,2,\text{…},m_{\ell }$, let

(61) $$C_{\ell }^{i}:=\left\{x\in R_{+}^{n}|\left\langle a_{\ell }^{i},x\right\rangle \leq \left(1+\beta_{\ell }\right)b_{i}^{\ell }\right\},$$

for all ℓ∈{1,2,…,p} where $R_{+}^{n}$ is the nonnegative orthant, and

(62) $$C_{\ell }^{i}:=\left\{x\in R_{+}^{n}|\left(1-\beta_{\ell }\right)c_{i}^{\ell }\leq \left\langle a_{\ell }^{i},x\right\rangle \right\},$$

for all ℓ∈{p+1,p+2,…,p+r}. Additionally, let

(63) $$Q_{\ell }:=\{A_{\ell }x=v\in R^{m_{\ell }}|∥{\left(v-b^{\ell }\right)}_{+}{∥}_{0}\leq \alpha_{\ell }m_{\ell }\},$$

for all $\ell \in \left\{1,2,\text{…},p\right\}\cap \bar{\Gamma }$ and

(64) $$Q_{\ell }:=\{A_{\ell }x=v\in R^{m_{\ell }}|∥{\left(c^{\ell }-v\right)}_{+}{∥}_{0}\leq \alpha_{\ell }m_{\ell }\}$$

for all $\ell \in \left\{p+1,p+2,\text{…},p+r\right\}\cap \bar{\Gamma }$. The above $A_{\ell }$ are blocks of the original matrix A and we denote by $A_{\ell }x=v$ the image of the vector x under $A_{\ell }$.

Problem 12 (**Translated problem of MOSCFPP for RTTP**) Let the operators $P_{C_{\ell }^{i}}:R^{n}\to R^{n}$ be orthogonal projections onto $C_{\ell }^{i}$ for all ℓ∈{1,2,…,p+r} and $i\in \{1,2,\text{…},m_{\ell }\}$, and let $P_{Q_{\ell }}:R^{m_{\ell }}\to R^{m_{\ell }}$ be orthogonal projections onto $Q_{\ell }$, for all ℓ∈Γ. The translated MOSCFPP for RTTP is as follows:

(65) $$\begin{array}{cc} \text{Find}\; & \text{an}\;x^{*}\in R_{+}^{n}\;\text{such}\;\text{that}\;x^{*}\in \cap_{\ell =1}^{p+r}\cap_{i=1}^{m_{\ell }}\mathrm{Fix}\left(P_{C_{\ell }^{i}}\right)\;\text{and,}\;\\ & \text{for}\;\text{all}\;\ell \in \Gamma ,\;A_{\ell }x^{*}\in \mathrm{Fix}\left(P_{Q_{\ell }}\right). \end{array}$$

We seek a solution to Problem 12 using our dynamic SA CQ‐method, described in Algorithm 1. We define, for all ℓ∈Γ,

(66) $$V_{\ell }:=Id-\gamma_{\ell }A_{\ell }^{T}\left(Id-P_{Q_{\ell }}\right)A_{\ell },$$

where $\gamma_{\ell }\in \left(0,\frac{1}{L_{\ell }}\right)$, $L_{\ell }={∥A_{\ell }∥}^{2}$ and $A_{\ell }^{T}$ is the transpose of $A_{\ell }$.

Remark 2 In practical use relaxation parameters play an important role:

(i) Each projection operator $P_{C_{\ell }^{i}}:R^{n}\to R^{n}$ may be relaxed with a parameter $\lambda_{\ell }\in \left(0,2\right)$ defined on the block ℓ∈{1,2,…,p+r}.

(ii) The relaxation parameters $\lambda_{\ell }$, as defined in (i), and $\gamma_{\ell }$, as given in (66), are permitted to take any value within their bounds on any iterative step of Algorithm 1. That is, they may depend on (vary with) the iteration index k and, therefore, be labeled $\lambda_{\ell }^{k}$ and $\gamma_{\ell }^{k}$.

(iii) The sets $Q_{\ell }$ are nonconvex and if for a given $\alpha_{\ell }m_{\ell }$ it is nonempty, then it is also closed and then projection onto $Q_{\ell }$ exists, is not necessarily unique, but can be calculated explicitly; see, for example, Penfold et al. (2017, Equation (24)). For properties regarding similar sets, see, for example, Beck (2017, Subsection 6.8.3). A recent work of Hesse et al. (2014) includes an investigation of these questions; see Section III there. Answers about the sets $Q_{\ell }$ and projections onto them in the specific setting related to the RTTP problem considered here are not yet available.

Tracking the percentage of elements in the current iteration of dose vectors $A_{\ell }x^{k}$ that are violating their constraints enables one to impose an adaptive version of Algorithm 1 using the comments in Remark 2. If, for example, one block has more PVC violations than LFP (dose limit constraints) violations then one could choose to alter the relaxation parameters at the next iteration, $\lambda_{\ell }^{k+1}$ and $\gamma_{\ell }^{k+1}$, in order to place less emphasis on the projections onto $C_{\ell }^{i}$.

## 8 Numerical implementation

### 8.1 Operator definitions

In Problem 12 we introduced the orthogonal projection operators $P_{C_{\ell }^{i}}$, which acts in the space of the pencil beam intensity vector x, and $P_{Q_{\ell }}$, which acts in the space of the dose vector $A_{\ell }x$. Here we provide explicit formulae, as examples, for calculating these projections in practice. Given an arbitrary vector $z\in R^{n}$ and some ℓ∈{1,2,…,p+r} and $i\in \{1,2,\text{…},m_{\ell }\}$, if it is the case that z is not in $C_{\ell }^{i}$ then it must be projected onto the nearest hyperplane which defines the boundary of $C_{\ell }^{i}$. Otherwise, no action is taken. If block ℓ represents an avoidance structure (ℓ∈{1,2,⋯,p}) then the projection can be calculated by

(67) $$P_{C_{\ell }^{i}}\left(z\right)=\left\{\begin{array}{cc} z, & \left\langle a_{\ell }^{i},z\right\rangle \leq \left(1+\beta_{\ell }\right)b_{i}^{\ell },\\ z+\lambda_{\ell }\frac{\left(1+\beta_{\ell }\right)b_{i}^{\ell }-\left\langle a_{\ell }^{i},z\right\rangle }{\left\langle a_{\ell }^{i},a_{\ell }^{i}\right\rangle }a_{\ell }^{i}, & \left\langle a_{\ell }^{i},z\right\rangle >\left(1+\beta_{\ell }\right)b_{i}^{\ell }, \end{array}\right.$$

where $\lambda_{\ell }\in \left(0,2\right)$ is a user‐selected relaxation parameter. Alternatively, if ℓ represents a target structure (ℓ∈{p+1,p+2,⋯,p+r}) then the projection can be similarly calculated using

(68) $$P_{C_{\ell }^{i}}\left(z\right)=\left\{\begin{array}{cc} z, & \left\langle a_{\ell }^{i},z\right\rangle \geq \left(1-\beta_{\ell }\right)c_{i}^{\ell },\\ z+\lambda_{\ell }\frac{\left(1-\beta_{\ell }\right)c_{i}^{\ell }-\left\langle a_{\ell }^{i},z\right\rangle }{\left\langle a_{\ell }^{i},a_{\ell }^{i}\right\rangle }a_{\ell }^{i}, & \left\langle a_{\ell }^{i},z\right\rangle <\left(1-\beta_{\ell }\right)c_{i}^{\ell }. \end{array}\right.$$

Note that, since in the above $\lambda_{\ell }\in \left(0,2\right)$ are used, the projections $P_{C_{\ell }^{i}}\left(z\right)$ are *relaxed* projections.

It is of interest to note that in clinical practice a structure may well have both an upper bound and a lower bound placed on the permitted dose. Such cases can be handled by simply defining two blocks for the same structure, one as an avoidance block, to which (67) applies, and one as a target block, to which (68) applies.

Projection of the dose vector onto $Q_{\ell }$ follows a slightly more elaborate procedure. We first define a helper set,

(69) $${\bar{Q}}_{\ell }:=\{y\in R^{m_{\ell }}|∥y_{+}{∥}_{0}\leq \alpha_{\ell }m_{\ell }\},$$

and describe the projection onto the set, $P_{{\bar{Q}}_{\ell }}$, by the following rules: for an arbitrary vector $y\in R^{m_{\ell }}$, first count the number of positive entries, $∥y_{+}{∥}_{0}$. If $∥y_{+}{∥}_{0}\leq \alpha_{\ell }m_{\ell }$ then the vector is in ${\bar{Q}}_{\ell }$ and no action is needed; $P_{{\bar{Q}}_{\ell }}=Id$, the identity operator. However, if $∥y_{+}{∥}_{0}>\alpha_{\ell }m_{\ell }$ then $P_{{\bar{Q}}_{\ell }}$ replaces the $\lfloor (∥y_{+}{∥}_{0}-\alpha_{\ell }m_{\ell })\rfloor$ smallest positive components of y with zeros and leaves the others unchanged. We can now define $P_{Q_{\ell }}$ in terms of a projection onto the helper set. Given $v\in R^{m_{\ell }}$,

(70) $$P_{Q_{\ell }}\left(v\right)=\left\{\begin{array}{cc} P_{{\bar{Q}}_{\ell }}\left(v-b^{\ell }\right)+b^{\ell }, & \ell \in \left\{1,2,\text{…},p\right\}\cap \bar{\Gamma },\\ -P_{{\bar{Q}}_{\ell }}\left(c^{\ell }-v\right)+c^{\ell }, & \ell \in \left\{p+1,p+2,\text{…},p+r\right\}\cap \bar{\Gamma }. \end{array}\right.$$

Since the sets ${\bar{Q}}_{\ell }$ are nonconvex, the projection is not necessarily unique. If this happens then any one of the possible vectors has to be chosen. The reader is referred to related results by Lu and Zhang (2012, Proposition 3.1), Hesse et al. (2014, Equation (20)), and Schaad (2010, p. 54).

### 8.2 Inverse planning algorithm

We provide here a practical example of how Algorithm 1 may be implemented for inverse planning in RTTP. In this example we initialize each of the beamlet weights to unit intensity, $x^{0}={\left(1,1,\cdots ,1\right)}^{T}$, before running through multiple cycles of an iterative scheme that is equivalent to a fully sequential Algorithm 1 with unit weights, $w_{k}=1$ for all k, in (33). The pseudo‐code of this procedure is detailed in Algorithm 2. The two “for” loop control cycles therein imply that the blocks, ℓ, may be chosen in any order, without replacement, and so may the voxels, i, within each block. Within each cycle, a nonnegativity constraint is enforced after all possible projections have been applied. This sets any unphysical negative entries in the beamlet intensity vector, x, to zero. In this example, a preset number of cycles are performed before stopping and accepting the final solution. However, one may easily replace this by a tolerance‐based stopping criterion.

Algorithm 2 **The dynamic SA CQ‐method: A pseudo‐code example for RTTP**

**Table:** 

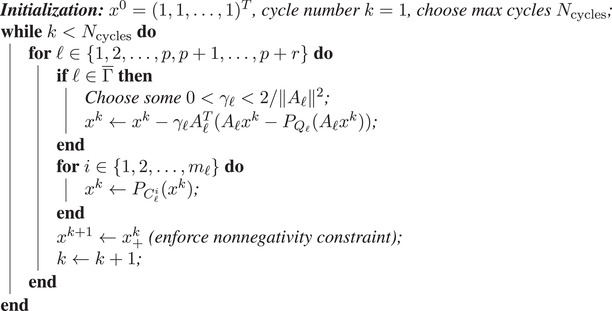

### 8.3 Numerical example

**Fig. 1:**
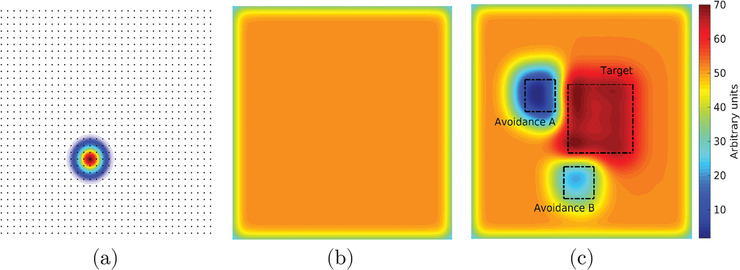
(a) A single Gaussian pseudo‐dose kernel contribution shown at one grid point. (b) Homogeneous pseudo‐dose of 50 units formed by superimposing all 1,156 Gaussian contributions. (c) Optimized pseudo‐dose map showing the structures for which the prescription in Table 1 was applied.

A two‐dimensional pseudo‐dose grid was created using *MATLAB*, version R2019a (The MathWorks, Inc., 2020). The grid is made of a matrix of dimensions 512×512 representing 262,144 pixels which altogether comprise an area of dosimetric interest. In a clinical treatment plan this would be the entire patient geometry and the pixels would be replaced by a large number of three‐dimensional voxels. Without loss of generality, we assume two spatial dimensions for simplicity. In order to achieve a basic emulation of dose deposited by multiple beamlets, 1156 Gaussian pseudo‐dose kernels were uniformly distributed across the grid. Each kernel had a standard deviation of 20 pixels and an amplitude such that their sum produced a homogeneous intensity map, with a mean value of 50 units. Figure 1a shows a visualization of the intensity (pseudo‐dose) matrix due to a single Gaussian kernel, with each dotted grid point representing the center of one of the 1156 kernels. Figure 1b shows the sum of all contributions. Note that each kernel contributes equally to the sum at this stage, prior to the inverse planning procedure. From this point on, for the proper RTTP context, we will assume that pixel values directly correspond to “dose.”

We have thus far introduced 1156 different matrices of dimensions 512×512. In order to form a *dose‐influence* matrix, A, for use in inverse planning, each matrix is collapsed to a single column vector with 262,144 entries, ensuring to keep track of which indices corresponded to which spatial positions in the dose grid. The matrix A is formed by all column vectors and therefore has 262,144 rows and 1156 columns.

A prescription composed of four HDCs, for minimum and maximum dose bounds, and three DVCs, shown in Table 1, was applied to three arbitrarily defined disjoint square regions. DVCs in Table 1 are written in the standard notation, $D_{V\%}$, which is the dose that is received by exactly V% of the structure. In the framework of this paper, an upper DVC on block ℓ is equivalent to writing $D_{100\alpha_{\ell }\%}\leq b^{\ell }$ and a lower DVC is equivalent to $D_{100\alpha_{\ell }\%}\geq c^{\ell }$. $D_{\max}$ and $D_{\min}$ represent the maximum and minimum dose constraints, respectively. The three defined square regions can be seen overlaying the dose solution in Fig. 1c. These consist of two avoidance regions, “Avoidance A” and “Avoidance B,” and one target region, “Target.” The column indices of the matrix A corresponding to pixels inside the boundary of these regions can be used to form submatrices, $A_{1}$, $A_{2}$, and $A_{3}$, respectively.

**Table 1:** Prescription chosen for the two‐dimensional numerical example

Structure	HDCs	DVCs
Avoidance A	$D_{\max}=25$	$D_{10}\%\leq 20$
Avoidance B	$D_{\max}=40$	$D_{25}\%\leq 30$
Target	$D_{\min}=60$	$D_{90}\%\geq 65$
	$D_{\max}=70$	

*Note*: Pseudo‐dose units are arbitrary. $D_{V\%}$ represents the dose that is received by exactly *V*% of the structure. $D_{\max}$ and $D_{\min}$ represent the maximum and minimum dose constraints, respectively.

We now have a framework in which Algorithm 2 can be applied. We have $A_{\ell }$ for ℓ∈{1,2,3} with p=2 and r=1, and we have $x^{0}={\left(1,1,\cdots ,1\right)}^{T}$ with 1156 entries. In this particular case, both lower and upper bounds on the dose have been prescribed for the “Target” structure. Therefore, we will actually use $A_{\ell }$ for ℓ∈{1,2,3,4}, where $A_{4}=A_{3}$ and ℓ=3 corresponds to the minimum dose constraint while ℓ=4 corresponds to the maximum dose constraint.

**Fig. 2:**
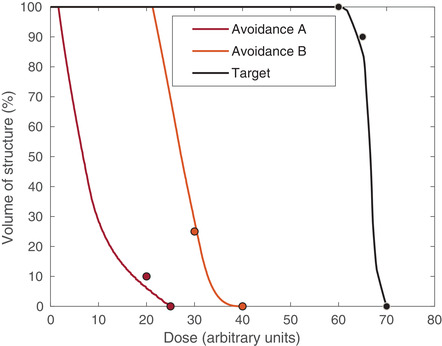
Cumulative dose‐volume histogram (DVH) showing the percentage of each structure that has received a certain dose. HDC and DVC prescriptions are shown as filled circles.

**Fig. 3:**
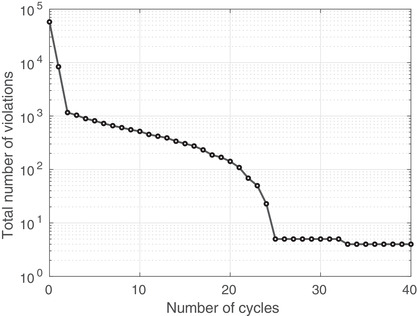
The number of total violations as a function of the number of the algorithmic cycles. A decrease indicates improvement in meeting the prescription.

**Fig. 4:**
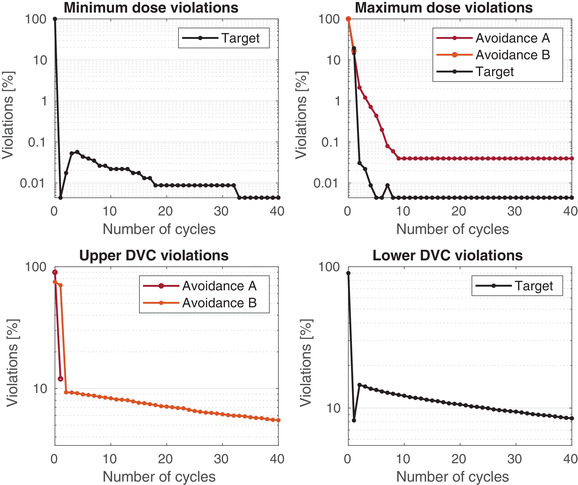
Percentage of violations as a function of the number of algorithmic cycles, shown separately for HDCs (minimum and maximum doses) and DVCs. An upper DVC is that which is applied to an avoidance structure while a lower DVC is that which is applied to a target structure.

Algorithm 2 was applied to the problem described above in order to reduce the dose in the avoidance structures and elevate it in the target structure, according to the prescription in Table 1. Forty cycles ($N_{\mathrm{cycles}}=40$) were used and the relaxation parameters, $\lambda_{\ell }$ and $\gamma_{\ell }$, were set to their midrange values, 1 and $1/∥|A_{\ell }{∥}^{2}$, respectively. Explicitly, $\lambda_{1}=\lambda_{2}=\lambda_{3}=\lambda_{4}=1$, $\gamma_{1}=1.546\times 10^{-6}$, $\gamma_{2}=1.545\times 10^{-6}$, and $\gamma_{3}=\gamma_{4}=1.030\times 10^{-6}$. Figure 1c shows a visualization of the dose solution following the algorithmic procedure. It is common in the clinic to evaluate plans using their dose‐volume histogram (DVH), which shows the percentage of each structure that has received a certain dose. Figure 2 shows a suitable DVH for this plan, with all prescriptions being approximately met. General convergence to the solution is indicated by a decrease in the total number of pixels violating the constraint imposed upon them, shown in the log‐loss plot in Fig. 3. Further, log‐loss plots for all four types of constraints (minimum dose, maximum dose, lower DVC, and upper DVC) are displayed in Fig. 4. Again, these all show a general decrease in the number of violations and, therefore, indicate that the solution gradually improves as the number of cycles increases.

As mentioned in Section 1.3, more extensive analysis in the context of RTTP and, in particular, medical physics is necessary in order to justify the use of the proposed dynamic SA CQ‐method. This work is ongoing and will be published in an appropriate medical physics journal.

## 9 Conclusions

We introduced a new split feasibility problem called MOSCFPP. This problem generalizes some well‐known split feasibility problems such as the split CFP, SCFPP, and more. Following the recent work of Penfold et al. (2017), and motivated from the field of RTTP, the MOSCFPP involves additional so‐called PVCs that give rise to nonconvex constraints sets. A new SA CQ‐method for solving the problem is introduced, which provides the user great flexibility in the weighting and order in which the projections onto the individual sets are executed.

**Table:** 

**List of acronyms**	
CFP	convex feasibility problem
CMSSCFP	constrained multiple set split convex feasibility problem
DVC	dose‐volume constraint
DVH	dose‐volume histogram
FNE	firmly nonexpansive
HDC	hard dose constraint
LFP	linear feasibility problem
MOSCFPP	multiple‐operator split common fixed point problem
MSA	modular string averaging
MSSCFP	multiple sets split convex feasibility problem
PVC	percentage violation constraint
RTTP	radiation therapy treatment planning
SCFP	split convex feasibility problem
SCFPP	split common fixed points problem
SIP	split inverse problem
SQNE	strongly quasi‐nonexpansive
